# *Xanthium strumarium* as an Inhibitor of α-Glucosidase, Protein Tyrosine Phosphatase 1β, Protein Glycation and ABTS^+^ for Diabetic and Its Complication

**DOI:** 10.3390/molecules21091241

**Published:** 2016-09-16

**Authors:** Seung Hwan Hwang, Zhiqiang Wang, Ha Na Yoon, Soon Sung Lim

**Affiliations:** Department of Food Science and Nutrition, Hallym University, 1 Hallymdeahak-gil, Chuncheon 24252, Korea; isohsh@gmail.com (S.H.H.); wangzq01234@gmail.com (Z.W.); yhnlove0219@naver.com (H.N.Y.)

**Keywords:** *Xanthium strumarium*, methyl-3,5-di-caffeoyquinic acid, protein tyrosine phosphatase 1β, α-glucosidase, advanced glycation end products

## Abstract

Phytochemical investigation of the natural products from *Xanthium strumarium* led to the isolation of fourteen compounds including seven caffeoylquinic acid (CQA) derivatives. The individual compounds were screened for inhibition of α-glucosidase, protein tyrosine phosphatase 1β (PTP1β), advanced glycation end products (AGEs), and ABTS^+^ radical scavenging activity using in vitro assays. Among the isolated compounds, methyl-3,5-di-caffeoyquinic acid exhibited significant inhibitory activity against α-glucosidase (18.42 μM), PTP1β (1.88 μM), AGEs (82.79 μM), and ABTS^+^ (6.03 μM). This effect was marked compared to that of the positive controls (acarbose 584.79 μM, sumarin 5.51 μM, aminoguanidine 1410.00 μM, and trolox 29.72 μM respectively). In addition, 3,5-di-*O*-CQA (88.14 μM) and protocatechuic acid (32.93 μM) had a considerable inhibitory effect against α-glucosidase and ABTS^+^. Based on these findings, methyl-3,5-di-caffeoyquinic acid was assumed to be potentially responsible for the anti-diabetic actions of *X. strumarium*.

## 1. Introduction

Diabetes mellitus and its related complications are some of the most prevalent and serious metabolic disorders characterized by high blood-glucose levels. One of the therapeutic approaches for counteracting hyperglycemia is to block the absorption of glucose by inhibiting the activity of carbohydrate-hydrolyzing enzymes such as α-glucosidase in the digestive organs. Small intestinal α-glucosidases (EC 3.2.1.20) are key enzymes involved in dietary carbohydrate digestion in humans [1]. Inhibitors of these enzymes may be effective in decreasing carbohydrate digestion and glucose absorption to suppress postprandial hyperglycemia [2]. However, α-glucosidase inhibitors (acarbose, miglitol and voglibose) have been shown to exhibit many side effects. A known α-glucosidase inhibitor, acarbose, is known to cause side effects such as abdominal pain, distension and diarrhea [3]. For this reason, many researchers have been trying to find a safe, potent and non-toxic α-glucosidase inhibitor from natural sources.

Protein tyrosine phosphatases (PTPases) are expressed in insulin-sensitive tissues (such as the liver, muscle and adipose tissue) and have a key role in the regulation of insulin signal transduction pathways [4]. Therefore, it is considered a favorable target for the treatment of type 2 diabetes mellitus (T2DM) and obesity [5]. Although several PTPases such as PTP-α, leukocyte antigen-related tyrosine phosphatase (LAR) and SH2-domain-containing phosphotyrosine phosphatase (SHP2) have been implicated in the regulation of insulin signaling, there is substantial evidence supporting PTP1β as the critical PTP controlling the insulin signaling pathway. PTP1β can interact with and dephosphorylate the activated insulin receptor (IR) as well as insulin receptor substrate (IRS) proteins [6].

Advanced glycation end products (AGEs) are formed from the non-enzymatic glycation of reducing sugars with the amino group of proteins. The subsequent products are Schiff bases that are then rearranged to more stable ketoamines [7]. It was proposed that accelerated chemical modification of proteins by glucose during hyperglycemia contributes to the pathogenesis of diabetes and the formation and accumulation of AGEs will induce oxidative stress and affect extracellular and intracellular function structure in many different cell types [8]. Therefore, AGEs are associated to increased oxidative stress and it would have deleterious effects on various cellular functions, and is thought to contribute to the pathogenesis of various diabetic complications (neuropathy, nephropathy, and retinopathy) [9].

The fruit of *Xanthium strumarium* has been used in traditional medicine in Korea for the treatment of chronic rhinitis, headache, cough and atopic dermatitis [10]. It has also been reported to possess curative effects against inflammation, allergic rhinitis, infection and cancer [11]. Various compounds have been isolated from this plant including caffeoylquinic acid (CQA) derivatives, carboxyatractyloside, xanthanol, isoxantanolm hydroquinone, alkaloids and thiazinedione [12]. Recently, our research team reported that CQA derivatives and phenolic constituents were isolated from the MeOH extract of *X. strumarium*. Furthermore, these compounds showed a strong inhibitory effect on aldose reductase [13]. Therefore, we investigated the inhibitory effect of *X. strumarium* on α-glucosidase, PTP1β, AGEs and ABTS^+^ to evaluate its potential for the prevention and treatment of diabetes mellitus.

## 2. Results

The purpose of this research is to investigate the inhibitory effects of *X. strumarium* on α-glucosidase, PTP1β, AGEs, and ABTS^+^ in vitro, and to isolate and analyze the α-glucosidase, PTP1β, AGEs, and ABTS^+^ from *X. strumarium*.

### 2.1. Structural Determination of Isolate Compounds

**Figure 1:**
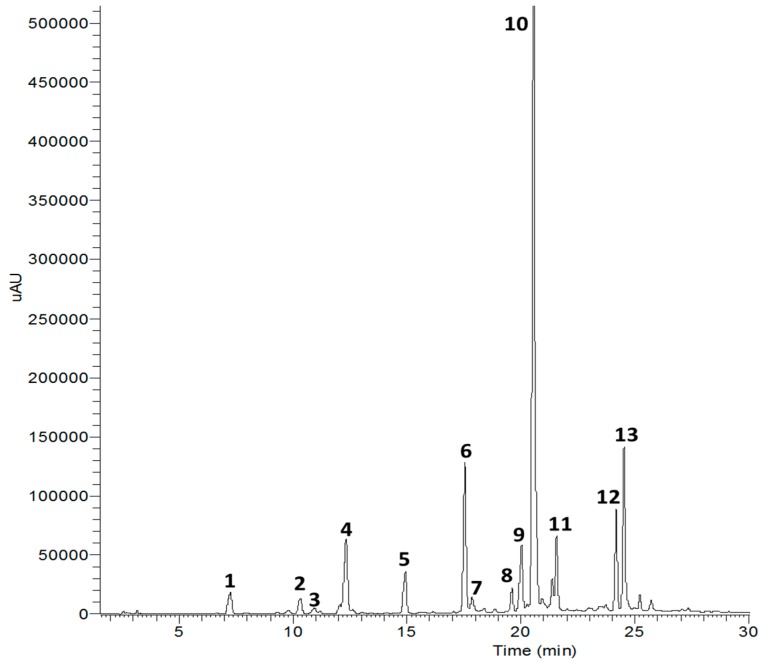
High performance liquid chromatography (254 nm) of compounds isolated from *X. strumarium*.

**Figure 2:**
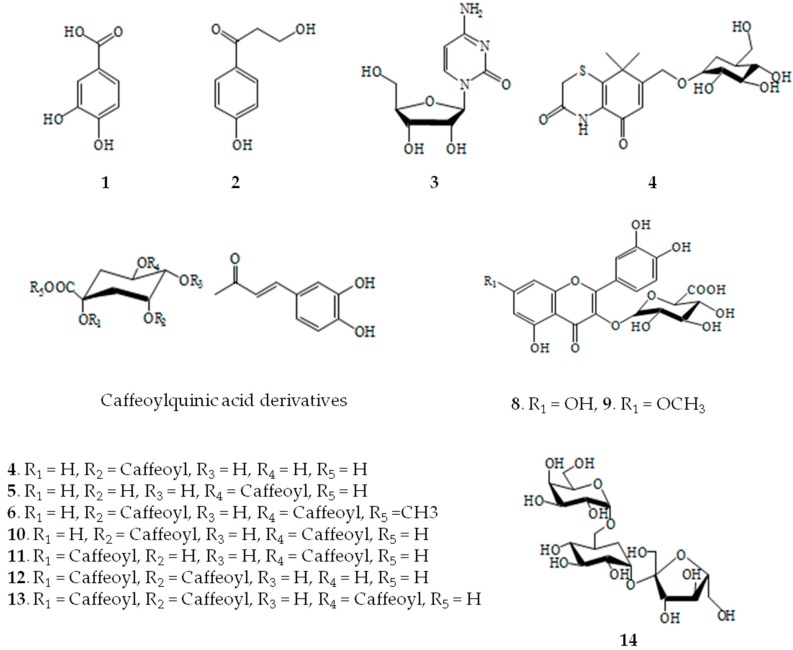
Structures of compounds isolated from X. *strumarium*.

Fourteen compounds were separated from EtOAc fraction by the RP C-18 column chromatography. These compounds were identified by comparing ^1^H- and ^13^C-NMR spectra and correlation NMR spectra such as correlation spectroscopy (COSY), heteronuclear multiple bond correlation (HMBC) and heteronuclear multiple quantum coherence (HMQC) with previously reported data and liquid chromatography-electrospray ionization-mass spectrometry (LC-ESI-MS) [13]. The fourteen compounds are compound **1** (protocatechuic acid), **2** (3-hydroxy-1-(4-hydroxy phenyl) propan-1-one), **3** (cytidine), **4** (neochlorogenic acid methyl ester), **5** (chlorogenic acid), **6** (methyl-3,5-di-*O*-caffeoylquinic acid), **7** (thiazine-3,5-dione-11-*O*-glucopyranoside), **8** (patuletin-3-glucuronide), **9** (quercetin-3-*O*-glucuronide), **10** (3,5-di-*O*-caffeoylquinic acid), **11** (1,5-di-*O*-caffeoylquinic acid), **12** (1,3-di-*O*-caffeoylquinic acid), **13** (1,3,5-tri-*O*-caffeoylquinic acid), and **14** (raffinose) (Figure 1 and Figure 2 and Table 1).

### 2.2. Inhibitory Effect of X. strumarium on α-Glucosidase

In order to identify the active compounds from *X. strumarium*, its extract was systematically partitioned into four fractions, which were then evaluated for α-glucosidase inhibitory effects (Table 2). Among the fractions, the EtOAc fraction (399.66 μg/mL) was found to exhibit a similar activity to the positive control, a known α-glucosidase inhibitor (377.19 μg/mL). The inhibitory activities of the isolated compounds **1**–**14** from *X. strumarium* against α-glucosidase were evaluated using acarbose (Table 3). Of the tested, compounds **6** and **10** showed strong inhibitory activity with IC_50_ values of 18.42 and 88.14 μM, respectively. However, other compounds had no an inhibitory effect even at the same concentration when compared to acarbose (584.79 μM).

### 2.3. Inhibitory Effects of X. strumarium on PTP1β

*X. strumarium* extract and the fourteen compounds were analyzed in vitro to investigate their inhibitory effects against PTP1β. All of the extracts and fractions showed significant inhibitory activity with IC_50_ values ranging from 9.80 to 28.44 μg/mL, compared with the positive control (8.96 μg/mL in Table 2). Of the fourteen compounds, compound **6** showed the most potent inhibitory activity, with an IC_50_ value of 1.88 μM, compared to the positive control sumarin (5.51 μM). However, other compounds had no inhibitory effect on PTP1β (Table 4).

### 2.4. Inhibitory Effects of X. strumarium on AGEs

The extract from *X. strumarium* and fractions were sequentially partitioned with water, CH_2_Cl_2_, EtOAc and *n*-BuOH. Each fraction was evaluated for AGEs using a bovine serum albumin-methylglyoxal assay. EtOAc and *n*-BuOH fractions exhibited potent inhibitory activity against AGE formation with IC_50_ values of 132.95 and 168.75 μg/mL respectively, compared with the positive control (166.22 μg/mL; Table 2). Since these results suggest the presence of AGEs inhibitors in the EtOAc fraction, special attention was focused into isolating the active constituent from this fraction. The inhibitory effects of isolated compounds **1**–**14** from EtOAc fractionare shown in Table 5. Among the isolated compounds, compound **6** (82.79 μM) exhibited significant inhibitory activity against methylglyoxal-mediated AGEs compared to the positive control AG (1410.00 μM). Conversely, CQA derivatives and other compounds displayed no inhibitory activity against AGEs.

### 2.5. Antioxidant Effect of *X*. strumarium on ABTS^+^

The antioxidant activities of the fractions and the constituents were evaluated in vitro by examining the ABTS^+^ radical scavenging activity and the results are summarized in Table 2. As shown in Table 2, the extract and fractions of *X. strumarium* exhibited strong inhibitory activity against ABTS^+^ (4.68–84.47 μg/mL) compared to the positive control trolox, which had an IC_50_ value of 7.51 μg/mL. Among the isolated compounds from the EtOAc fraction, compound **6** had the strongest inhibitory activity with an IC_50_ value of 6.03 μM and compound **1** also exhibited potent inhibitory activity with an IC_50_ value of 32.93 μM compared to trolox (29.72 μM, Table 6). The fourteen compounds isolated from *X. strumarium* were evaluated using AGEs, PTP1β, α-glucosidase and ABTS^+^ assays. Among the CQA derivatives, compound **6** was found to be an effective inhibitor of α-glucosidase, PTP1β, AGEs, and ABTS^+^. It exhibited 31.74-, 2.93-, 17.03-, and 4.93- fold higher inhibition than the positive controls acarbose, sumarin, aminoguanidine, and trolox, respectively. Additionally, compound **10** had an IC_50_ value of 88.14 μM for α-glucosidase compared to the positive control acarbose (584.79 μM).

## 3. Discussion

*X. strumarium* is a traditional herb medicine and its ethnomedicinal uses including to control blood sugar in diabetic patients. EzazulHaque et al. has reported the antihyperglycemic activity of *X. strumarium* in vivo [14].

The compound **6**, isolated from *X. strumarium*, is an ester derivative which is difference from the other compounds. Of the tested compounds, **6**, which contains a methyl ester at quinic acid moiety, exhibited the most potent inhibitory activity with an IC_50_ value of 82.79 μM on AGEs, 1.88 μM on PTP1β, 18.42 μM on α-glucosidase, and 6.03 μM on ABTS^+^, respectively. The compound **10** only exhibited α-glucosidase inhibitory activity, whereas compounds **11**–**13**, which include two/three caffeoyl groups and different position at quinic acid moiety, showed no inhibitory activity against AGEs, PTP1β, and ABTS^+^, respectively. The present study demonstrated that the methyl esters of the isolates were stronger inhibitors of anti-diabetic implying the importance of esterification for inhibitory potency.

Previous investigations into the anti-diabetic effects of CQA derivatives by Chen et al. reported that 3,4-di-*O*-CQA (187.2 μM), 4,5-di-*O*-CQA (130.8 μM), methyl 3,4-di-*O*-CQA (12.23 μM), and methyl 4,5-di-*O*-CQA (13.08 μM) isolated from the aerial parts of *Gynura divaricate* showed the inhibitory effects against α-glucosidase and compared to that of the positive control (acarbose 867.4 μM). This literature indicated that the inhibitory activities of methyl-CQA derivatives were almost ten times higher than CQA derivatives on α-glucosidase. In addition, Chen et al. also was suggested that addition of a methyl group to the quinic acid skeleton may be responsible for a loss of α-glucosidase inhibitory activity [15]. In another research, Hwang et al. reported that 3′-methoxyhirsutrin isolated from *Zea mays* L. showed inhibitory activity with IC_50_ value of 64.04 μM. Whereas, hirsutrin isolated from the same plant showed no activity on inhibition of PTP1β. These results demonstrated that the methyl group on the polyphenol skeleton may also play an important role contribute to the loss of PTP1β inhibitory activity [16]. In addition, compound **6** and caffeic acid methyl ester isolated from highbush blueberry fruit have been shown to exhibit α-glucosidase [17] and compound **6** isolated from the extracts of *Erigeron annuus* also showed the strong inhibitory activity on AGEs [18].

3-*O*-CQA, 3,4-di-*O*-CQA, compound **10**, and 3,4,5-tri-*O*-CQA isolated from Brazilian propolis possessed α-glucosidase and α-amylase inhibitory activities [19]. In addition, 3-*O*-CQA, 4-*O*-CQA, 5-*O*-CQA, and 3,5-di-*O*-caffeoyl-*epi*-quinic acid isolated from the extracts of *Erigeron annuus* and *Artemisia montana* exhibited the most potent inhibitory activity in both the AGEs and AR [18,20]. Compounds **6**, **10**, **11**, **12**, and **13** from *X. strumarium* exhibited strong rat lens and recombinant aldose reductase inhibitory activities [13]. Recent literature suggests that CQA has physiological properties; 3,5-dicaffeoyl-4-succinylquinic and compound **10** from *Chrysanthemum coronarium* have anti-oxidant activities [21]. Further, anti-inflammatory effects are exhibited by 3,4-di-*O*-CQA methyl ester, compounds **6** and **10** isolated from *Ligularia* *fischeri* leaves and *Ilex latifolia* [22,23].

There are several widely used drug development targets for treatment of hyperglycemia, including PTP1β and α-glucosidase. And therapeutic measures for the treatment of hyperglycemic include the use of PTP1β inhibitors and α-glucosidase inhibitors. α-Glucosidase are a group of key intestinal enzymes involved in the digestion of carbohydrates and α-glucosidase inhibitors can be used to delay the absorption of carbohydrates from small intestine and thus lower postprandial blood glucose [24]. PTP1β is a major non-trans membrane phosphotyrosine phosphatase in human tissues and was one of the earliest PTP identified. Although its physiological function and mechanism of regulation are largely unknown, it has been demonstrated to dephosphorylate insulin receptor in intact cells and thus to act as a negative regulator of insulin signaling. Moreover, the deletion of PTP1β gene in mice caused marked insulin sensitivity and prolonged insulin receptor auto-phosphorylation. PTP1β inhibitor would increase insulin sensitivity by blocking the PTP1β-mediated negative insulin signaling pathway and thus lower postprandial blood glucose [25,26].

Our data suggests that there was no significant relationship between structure of CQA derivatives and their inhibitory activity. However, it is postulated that the number of caffeoyl groups and their positions on the quinic acid moiety may be important factors in conferring the inhibitory activity. Our results and past literature reported that CQA with two caffeic acid methyl groups is more effective in inhibiting α-glucosidase than that of two caffeic acid also combines three CQA showed activity similar to anything (compound **6** > compound **10** > 3,4-di-*O*-CQA). In contrast, AGEs and PTP1β had single CQA more inhibitory effect than that of two/three caffeic acid including methyl group.

## 4. Experimental Section

### 4.1. General Experimental Procedures

^1^H- and ^13^C-NMR spectra and correlation NMR spectra such as COSY, HMBC, HMQC, and DEPT were obtained from a Bruker Avance DPX 400 (or 600) spectrometer (Berlin, Germany). These were obtained at operating frequencies of 400 MHz (^1^H) and 100 (or 150) MHz (^13^C) with CD_3_OD, (CD_3_)_2_SO, (CD_3_)_2_CO, or D_2_O and TMS was used as an internal standard; chemical shifts were reported in δ values. The α-glucosidase from *Saccharomyces* sp. (SSG) was purchased from Wako Pure Chemical Industries Ltd (Tokyo, Japan). Acarbose, aminoguanidine, trolox, suramin, bovine serum albumin, methylglyoxal and *p*-nitrophenyl-α-glucopyranoside (*p*NPG) as a synthetic substrate were obtained from Sigma–Aldrich Co. (St. Louis, MO, USA). All other chemicals and reagents used were of analytical grade.

### 4.2. Plant Materials

Plant material “Chang-i-ja” used in this study was purchased from a local market in Chuncheon. The voucher sample (RIC-HU1204) has been deposited at the center for efficacy assessment and development of functional foods and drugs, Hallym University, Chuncheon.

### 4.3. Extraction and Isolation

Dried *X. strumarium* (4.5 kg) were ground and extracted with CH_2_Cl_2_ at room temperature. The residue was refluxed for 3 h with MeOH three times at room temperature and evaporated under reduced pressure to give a residue (165.0 g). The residue was suspended in distilled water and partitioned with CH_2_Cl_2_, EtOAc, *n*-BuOH and H_2_O successively to afford CH_2_Cl_2_ (7.4 g), EtOAc (25.2 g), *n*-BuOH (33.1 g) and H_2_O fraction (65.7 g). The EtOAc fraction showed strong inhibitory activity on AGEs, so this fraction (3.0 g) was subjected to RP C-18 column chromatography for further with MeOH gradient system (1:4 to 1:1) to yield compounds **1** (25.3 mg), **2** (12.8 mg), **3** (9.7 mg), **4** (12.4 mg), **5** (17.5 mg), **6** (4.8 mg), **7** (8.8 mg), **8** (11.88 mg), **9** (25.4 mg), **10** (5.8 mg), **11** (9.2 mg), **12** (4.2 mg), **13** (13.8 mg) and **14** (8.4 mg) (Figure 1).

### 4.4. Assay for the α-Glucosidase Inhibitory Activity

The α-glucosidase inhibitory activity of the extracts and fractions was determined using a modified procedure reported method with a slight modification [1]. The α-glucosidase activity was measured using the substrate *p*-nitrophenyl-α-d-glucopyranoside (*p*NPG), which is hydrolyzed by α-glucosidase to release the product *p*-nitrophenol, a colorant that can be monitored at 405 nm. The initial concentration of the enzyme solution was 0.62 unit/mL in 0.1 M phosphate buffer (pH 6.9) and the initial concentration of the substrate solution was 2 mM in the same phosphate buffer. The enzyme solution was mixed with water and the samples or controls in a clear 96-well microplate (flat bottom) and the reaction was initiated by addition of the substrate to the solution. The plates were incubated at 37 °C for 5 min and the reaction was terminated by the addition of 0.1 M Na_2_CO_3_. Enzyme inhibition was determined by the absorbance of 4-nitrophenol (product) at 405 nm, as measured with a microplate reader. Background absorbance was determined using a non-enzyme control microplate containing the buffer and was subtracted from the absorbance of the samples and controls. The concentration of inhibitors resulting in 50% inhibition of enzyme activity (IC_50_) was calculated from the least square regression line of the logarithmic concentrations plotted against the residual activity. Acarbose was used as positive control.

### 4.5. Assay for the PTP1β Inhibitory Activity

PTP1β tyrosine phosphatase drug discovery kit is a colorimetric, non-radioactive assay designed to measure the phosphatase activity of purified PTP1β. The enzyme activity was measured using IR5 phosphopeptide (insulin receptor B residues 1142–1153, pY-114) as a substrate. To each well of the 96-well microplate (final volume: 125 μL), 75 μM IR5 substrate and PTP1β (2.5 ng/well) in a buffer containing 100 mM MES (pH 6.0), 0.3 M NaCl, 2 mM EDTA, 2 m Mdithiothreitol (DTT) and 0.1% NP-40 were added with or without test compounds. Following incubation at 37 °C for 30 min, the reaction was terminated with the BIOMOL RED^TM^ reagent. The amount of *p*-nitrophenol produced was estimated by measuring the absorbance at 620 nm. The non-enzymatic hydrolysis of the IR5 substrate was corrected by measuring the increase in absorbance at 620 nm obtained in the absence of PTP1β enzyme.

### 4.6. Assay for the AGEs Inhibitory Activity

The modified procedure of Lee et al. was followed [9]. Bovine serum albumin (10 mg/mL) was incubated with 5 mM methylglyoxal in sodium phosphate buffer (100 mM; pH 7.4). Dimethylsulfoxide used for dissolving samples was found to have no effect on the reaction. All of the reagents and samples were dissolved by filtration through 0.2 μm membrane filters and the mixture was incubated at 37 °C for 7 days. The fluorescence intensity was measured at an excitation wavelength of 330 nm and an emission wavelength of 410 nm with a LS50B fluorescence spectrometer Perkin-Elmer Ltd., (Buckinghamshire, FLS, UK). Aminoguanidine was also tested as a known inhibitor.

### 4.7. Assay for the ABTS^+^ Inhibitory Activity

The method described by Li et al. was used with slight modifications [27]. ABTS^+^ diammonium salt (2 mM) and potassium persulfate (3.5 mM) were mixed, diluted in distilled water and kept in the dark at room temperature for 24 h before use. After addition of ABTS^+^ solution to 10 µL of antioxidant, measurements were recorded at 10 min post reaction. The percentage inhibition of absorbance at 750 nm was calculated and plotted as a function of concentration of antioxidants. Trolox was used as positive control.

## 5. Conclusions

In summary, among the CQA derivatives isolated from *X. strumarium*, our results suggest that *X. strumarium* and compound **6** are a potent inhibitor of α-glucosidase, PTP1β, AGEs, and ABTS^+^, in contributing at least in part for prevention and treatment of diabetes. The contribution of this research is to provide the fundamental knowledge for development of new α-glucosidase and PTP1β inhibitors from *X. strumarium* and/or its components. Finally, further our studies will need to more understand the functional mechanism of *X. strumarium* and its bioactive components.

## Figures and Tables

**Table 1:** Identification of the major compounds detected of *X. strumarium* by LC-ESI-MS.

Peaks	Rt (min)	UV/Vis λ_Max_ (nm)	LC-ESI-MS		Compounds
			Mass [M]^+^ (*m/z*)	Fragment [M + H]^+^ (*m/z*)	
1	7.20	294, 258, 227	154.12	137.58	Protocatechuic acid
2	10.28	290, 254, 229	166.17	108.14	3-Hydroxy-1-(4-hydroxy phenyl)propan-1-one
3	10.94	266, 219	243.22	111.10	Cytidine
4	12.18	328, 243	368.34	180.16	Neochlorogenic acid methyl ester
5	14.86	327, 242, 224	353.99	209.22	Chlorogenic acid
6	17.55	330, 244	530.48	368.34	Methyl-3,5-di-*O*-caffeoylquinic acid
7	17.90	346, 242	401.67	239.29	Thiazine-3,5-dione-11-*O*-glucopyranoside
8	19.34	352, 257, 223	508.71	332.26	Patuletin-3-glucuronide
9	20.13	354, 256, 202	478.36	302.24	Quercetin-3-*O*-glucuronide
10	20.59	328, 243, 223	516.17	354.20	3,5-Di-*O*-caffeoylquinic acid
11	21.51	330, 244, 223	516.17	354.20	1,5-Di-*O*-caffeoylquinic acid
12	24.18	328, 245, 223	516.17	354.20	1,3-Di-*O*-caffeoylquinic acid
13	24.78	327, 244, 224	678.27	516.45	1,3,5-Tri-*O*-caffeoylquinic acid

**Table 2:** Inhibitory effect of the crude extract and fractions of *X. strumarium* on α-glucosidase, protein tyrosine phosphatase 1β (PTP1β), advanced glycation end products (AGEs), and ABTS^+^.

Entry	Acarbose ^1^	Suramin ^2^	Aminoguanidine ^3^	Trolox ^4^	MeOH	CH_2_Cl_2_	EtOAc	*n*-BuOH	Water
	IC_50_ (μg/mL) ^5^								
	Positive Control				Extract	Fraction			
α-Glucosidase	377.19 ± 38.17	-	-	-	>500	-	399.66 ± 37.51	-	-
PTP1β	-	8.96 + 0.83	-	-	12.88 ± 1.18	20.81 ± 2.09	9.80 ± 0.89	15.08 ± 1.46	28.44 ± 2.79
AGEs	-	-	166.22 ± 16.29	-	>200	>200	132.95 ± 12.34	168.75 ± 17.87	-
ABTS^+^	-	-	-	7.51 ± 0.58	78.32 ± 0.71	8.52 ± 0.86	9.34 ± 0.92	84.47 ± 8.51	4.68 ± 4.61

^1^ Acarboseis the positive control for α-glucosidase. ^2^ Suramin is the positive control for protein tyrosine phosphatase 1β. ^3^ Aminoguanidine is the positive control for advanced glycation end products. ^4^ Trolox is the positive control for ABTS^+^ radical scavenging activity. ^5^ The IC_50_ value was defined as the half-maximal inhibitory concentration and mean of 3 duplication analyses of each sample. - is no activity.

**Table 3:** Inhibitory effect of isolated compounds from *X. strumarium* on α-glucosidase.

Entry	Products	Concentration (μg/mL)	Inhibition (%)	IC_50_^1^ (μg/mL)
	Acarbose ^2^	1000	106.14	377.19 ± 37.8 (584.79 μM)
		500	60.29	
		250	41.06	
		50	16.98	
1	Protocatechuic acid	50	17.45	-
2	3-Hydroxy-1-(4-hydroxy phenyl) propan-1-one	50	8.60	-
3	Cytidine	50	4.70	-
4	Neochlorogenic acid methyl ester	50	-	-
5	Chlorogenic acid	50	6.60	-
6	Methyl-3,5-di-*O*-caffeoylquinic acid	25	96.50	9.78 ± 0.79 (18.42 μM)
		12.5	59.11	
		5	40.00	
		2.5	22.63	
7	Thiazine-3,5-dione-11-*O*-glucopyranoside	50	10.18	-
8	Patuletin-3-glucuronide	50	6.11	-
9	Quercetin-3-*O*-glucuronide	50	15.40	-
10	3,5-di-*O*-caffeoylquinic acid	100	99.44	45.48 ± 4.52 (88.14 μM)
		50	52.85	
		25	33.72	
		5	9.34	
11	1,5-di-*O*-caffeoylquinic acid	50	46.20	-
12	1,3-di-*O*-caffeoylquinic acid	50	38.37	-
13	1,3,5-tri-*O*-caffeoylquinic acid	50	22.05	-
14	Raffinose	50	5.82	-

^1^ The IC_50_ value was defined as the half-maximal inhibitory concentration and mean of 3 duplication analyses of each sample. ^2^ Acarbose was used as the positive control. - is no activity.

**Table 4:** Inhibitory effect of isolated compounds from *X. strumarium* on protein tyrosine phosphatase 1β (PTP1β).

Entry	Products	Concentration (μg/mL)	Inhibition (%)	IC_50_^1^ (μg/mL)
	Sumarin ^2^	14.29	83.58	7.84 ± 0.75 (5.51 μM)
		7.15	52.24	
		3.57	26.44	
		1.43	10.45	
1	Protocatechuic acid	10	28.58	-
2	3-Hydroxy-1-(4-hydroxy phenyl) propan-1-one	10	5.88	-
3	Cytidine	10	-	-
4	Neochlorogenic acid methyl ester	10	18.79	-
5	Chlorogenic acid	10	31.34	-
6	Methyl-3,5-di-*O*-caffeoylquinic acid	10	82.47	5.09 ± 0.50 (1.88 μM)
		5	50.01	
		2.5	30.66	
		1	24.00	
7	Thiazine-3,5-dione-11-*O*-glucopyranoside	10	-	-
8	Patuletin-3-glucuronide	10	-	-
9	Quercetin-3-*O*-glucuronide	10	10.71	-
10	3,5-di-*O*-caffeoylquinic acid	10	18.40	-
11	1,5-di-*O*-caffeoylquinic acid	10	10.62	-
12	1,3-di-*O*-caffeoylquinic acid	10	22.34	-
13	1,3,5-tri-*O*-caffeoylquinic acid	10	39.17	-
14	Raffinose	10	-	-

^1^ The IC_50_ value was defined as the half-maximal inhibitory concentration and mean of 3 duplication analyses of each sample. ^2^ Sumarin was used as the positive control. - is no activity.

**Table 5:** Inhibitory effect of isolated compounds from *X. strumarium* on advanced glycation end products (AGEs).

Entry	Products	Concentration (μg/mL)	Inhibition (%)	IC_50_^1^ (μg/mL)
	Aminoguanidine ^2^	400	98.17	155.88 ± 15.45 (1410.00 μM)
		200	61.01	
		100	37.84	
		50	27.97	
1	Protocatechuic acid	100	9.49	-
2	3-Hydroxy-1-(4-hydroxy phenyl) propan-1-one	100	8.50	-
3	Cytidine	100	33.16	-
4	Neochlorogenic acid methyl ester	100	11.40	-
5	Chlorogenic acid	100	7.47	-
6	Methyl-3,5-di-*O*-caffeoylquinic acid	100	89.47	43.96 ± 3.89 (82.79 μM)
		50	54.77	
		20	34.59	
		10	24.24	
7	Thiazine-3,5-dione-11-*O*-glucopyranoside	100	22.24	-
8	Patuletin-3-glucuronide	100	6.11	-
9	Quercetin-3-*O*-glucuronide	100	23.96	-
10	3,5-Di-*O*-caffeoylquinic acid	100	42.91	-
11	1,5-Di-*O*-caffeoylquinic acid	100	25.04	-
12	1,3-Di-*O*-caffeoylquinic acid	100	40.19	-
13	1,3,5-Tri-*O*-caffeoylquinic acid	100	40.22	-
14	Raffinose	100	14.99	-

^1^ The IC_50_ value was defined as the half-maximal inhibitory concentration and mean of 3 duplication analyses of each sample. ^2^ Aminoguanidine was used as the positive control. - is no activity.

**Table 6:** Inhibitory effect of isolated compounds from *X. strumarium* on ABTS^+^ radical scavenging activity.

Entry	Products	Concentration (μg/mL)	Inhibition (%)	IC_50_^1^ (μg/mL)
	Trolox ^2^	16.66	95.06	7.43 ± 0.71 (29.72 μM)
		8.33	54.14	
		3.33	34.52	
		1.66	17.27	
1	Protocatechuic acid	16.66	97.22	4.94 ± 0.38 (32.93 μM)
		8.33	68.72	
		3.33	52.46	
		1.66	25.17	
2	3-Hydroxy-1-(4-hydroxy phenyl) propan-1-one	3.33	-	-
3	Cytidine	3.33	-	-
4	Neochlorogenic acid methyl ester	3.33	8.66	-
5	Chlorogenic acid	3.33	23.59	-
6	Methyl-3,5-di-*O*-caffeoylquinic acid	8.33	98.31	3.20 ± 0.28 (6.03 μM)
		3.33	69.25	
		1.66	33.69	
		0.33	7.49	
7	Thiazine-3,5-dione-11-*O*-glucopyranoside	3.33	-	-
8	Patuletin-3-glucuronide	3.33	30.53	-
9	Quercetin-3-*O*-glucuronide	3.33	12.32	-
10	3,5-Di-*O*-caffeoylquinic acid	3.33	40.74	-
11	1,5-Di-*O*-caffeoylquinic acid	3.33	30.48	-
12	1,3-Di-*O*-caffeoylquinic acid	3.33	22.12	-
13	1,3,5-Tri-*O*-caffeoylquinic acid	3.33	38.06	-
14	Raffinose	3.33	-	-

^1^ The IC_50_ value was defined as the half-maximal inhibitory concentration and mean of 3 duplication analyses of each sample. ^2^ Trolox was used as the positive control. - is no activity.
